# How Can the National Burden of Parkinson’s Disease Comorbidity and Mortality Be Estimated for the Japanese Population?

**DOI:** 10.2188/jea.JE20100149

**Published:** 2011-05-05

**Authors:** Yuriko Doi, Tetsuji Yokoyama, Yoshikazu Nakamura, Masaki Nagai, Kenichi Fujimoto, Imaharu Nakano

**Affiliations:** 1 Department of Education and Training Technology, National Institute of Public Health, Saitama, Japan; 2 Department of Human Resources Development, National Institute of Public Health, Saitama, Japan; 3 Department of Public Health, Jichi Medical University School of Medicine, Tochigi, Japan; 4 Department of Public Health, Saitama Medical University, Saitama, Japan; 5 Division of Neurology, Department of Medicine, Jichi Medical University School of Medicine, Tochigi, Japan

**Keywords:** Parkinson’s disease, comorbidity, mortality, causes of death, Japan

## Abstract

**Background:**

Good medical care results in long survival for patients with Parkinson’s disease (PD). However, little is known about the burden of PD comorbidity and mortality in Japan. This is the first study to examine comorbid diseases of PD decedents and extrapolate PD death rates from multiple-cause coding mortality data for the total population of Japan.

**Methods:**

Data for 4589 certified deaths due to PD as the underlying cause of death (ICD-10 code: G20) were obtained from the 2008 Japanese vital statistics. Of those, comorbidities listed in the death certificates of 477 randomly selected decedents were analyzed. All diseases or conditions mentioned on death certificates were counted and ranked in descending order of frequency. The death rates (per 100 000 population) from PD were calculated using Japanese National Vital Statistics. The estimated rate of deaths with PD was extrapolated using US death data from a multiple-cause coding system, as no such system is available in Japan, with adjustment for the difference in disease structure between countries.

**Results:**

Average age at death was 80.9 years. The top 5 comorbid diseases ranked as contributory causes of death were cerebrovascular diseases (4.0%), dementia (3.8%), diabetes mellitus (3.6%), malignant neoplasm (2.5%), and heart diseases (2.3%). Overall, the death rates from and with PD were 3.6 and 5.8, respectively.

**Conclusions:**

Analysis restricted to data from the underlying-cause coding system underestimated the national burden of PD comorbidity and mortality. Use of death certificates and multiple-cause mortality data complement the existing system.

## INTRODUCTION

Under the Statistics Act of Japan, the Japanese Ministry of Health, Labour and Welfare is charged with overseeing the annual collection of vital statistics surveys to analyze vital events and obtain a basic population data source for policy making on health, labor, and welfare.^1^ The procedures adhere to international standards for mortality statistics regarding underlying cause of death, which is defined by the World Health Organization as the disease or injury that initiated the train of morbid events leading directly to death or the circumstances of the accident or violence that produced the fatal injury.^2^ Coders select underlying causes of deaths in accordance with the rules and guidelines on coding for deaths and diseases.^3^ Overall, underlying cause of death data are reported to capture approximately 90% of deaths mentioned in the death certificates for malignant neoplasms.^4^^–^^8^ Statistics on underlying cause of death can be valuable in describing types of death for which a single primary cause is clinically considered to contribute.

Parkinson’s disease (PD) is a progressive neurodegenerative disorder characterized by the 4 cardinal motor signs of tremor at rest, bradykinesia, rigidity, and postural instability, and by other non-motor clinical manifestations. Average age at onset is approximately 55 years. With the development of various kinds of treatments, the average age at death of patients with PD is now close to that of the general population. Because the disease is incurable, patients survive for a long period, often decades, under medical supervision.^9^

As with other chronic diseases, the causes of PD death are more likely to represent a number of co-existing conditions among which there may be no direct etiologic chain to facilitate the identification of a single underlying cause. To complement statistics on underlying cause of death, statistics encompassing multiple causes of death have been introduced as standard practice in a number of Western countries. Analysis of multiple cause of death revealed that data on underlying cause of death represent only 30% to 50% of deaths with PD mentioned in death certificates.^4^^,^^8^^,^^10^ How can we estimate the national burden of PD comorbidity and mortality without a multiple-cause coding system for mortality statistics in Japan? The present study is the first to examine comorbid diseases of decedents from PD using their death certificates and to extrapolate PD death rates from multiple-cause coding mortality data.

## METHODS

### Data

#### Death certificates

There were 4589 Japanese decedents for whom PD was the underlying cause of death in the 2008 vital statistics. To analyze these death certificates, a random sample of decedents was selected, with a 10% probability for each prefecture. There were no significant differences in demographic characteristics between the vital statistics dataset and the sampled death certificates (Table 1).

**Table 1.** Demographic characteristics of decedents for whom Parkinson’s disease was listed as the underlying cause of death on death certificates (Japan, 2008)

Characteristics	All decedents		Sampled decedents^a^	
	(*n* = 4589)		(*n* = 477)	
	
	Number or Mean	% or SD	Number or Mean	% or SD
Sex				
Men	2124	46.3	208	43.6
Women	2465	53.7	269	56.4
Age^b^				
Men	79.5	7.2	80.1	7.2
Women	82.0	7.3	82.7	6.9
Both	80.9	7.4	81.6	7.1
Marital status				
Married	2380	51.9	239	50.1
Single	184	4.0	21	4.4
Bereaved	1865	40.6	200	41.9
Separated	158	3.4	17	3.6
Unknown	2	0.0	0	0.0
Occupation				
Farming	349	7.6	48	10.1
Self-employed	242	5.3	31	6.5
Employed	570	12.5	60	12.5
Other	302	6.6	29	6.1
Unemployed	2718	59.2	266	55.8
Unknown	408	8.9	43	9.0
Place of death				
Hospital	3496	76.2	363	76.1
Clinic	156	3.4	11	2.3
Healthcare ​ facility for the aged	103	2.2	15	3.1
Nursing home	325	7.1	33	6.9
Home	466	10.2	48	10.1
Other	43	0.9	7	1.5

^a^Sampled decedents were randomly selected with a 10% probability from all deaths attributed to Parkinson’s disease, by prefecture, in 2008.

^b^Values are means and standard deviations (SDs).

Two of the authors (YD and TY) are medical epidemiologists and transcribed all mentioned causes of death from the copied death certificates of the sampled decedents, after obtaining permission to do so from the Statistics and Information Department, Minister’s Secretariat, Ministry of Health, Labour and Welfare, Japan. The transcribed information did not contain any personally identifiable information. Based on standard clinical practice, one author (YD), who has more than 10 years of experience in clinical medicine, classified all mentioned causes of death other than PD into several categories of diseases and medical conditions. The present study was approved in March 2010 by the Institutional Review Board of the National Institute of Public Health, Japan.

#### Vital statistics

Data for 4589 decedents from PD as the underlying cause of death were extracted from the national mortality database of vital statistics, after obtaining permission from the Statistics and Information Department, Minister’s Secretariat, Ministry of Health, Labour and Welfare, Japan. The International Statistical Classification of Diseases and Related Health Problems 10th Revision (ICD-10) uses the code G20 for PD.^2^^,^^11^ The data used in the present study did not contain any personally identifiable information.

### Statistical analysis

#### Analysis of death certificates

All categorized causes or conditions mentioned, other than PD, in death certificates of the sampled decedents were counted and ranked in descending order of frequency as 1 of 3 types of causes: immediate, intermediate, or contributory. An immediate cause is a final disease or condition resulting in death and is described on the top line in Part (I) of the death certificate. Intermediate causes are diseases, injuries, or complications (other than immediate cause) in the chain of events that directly caused the death and are described in Part (I) of the death certificate. Contributory causes are other significant conditions that contributed to the causes described in Part (I), but did not directly result in those causes. Contributory causes are described in Part (II) of the death certificate.^12^

#### Analysis of vital statistics

The number and rate of deaths in individuals for whom PD was listed as the underlying cause of death were calculated according to 5-year age intervals using the Japanese national vital statistics and data on the Japanese population for the year 2008. Currently, there is no multiple-cause coding system in Japan; however, the US Centers for Disease Control and Prevention (CDC) publically releases data on multiple cause-of-death. Therefore, we calculated the number and rate of PD as a multiple cause of death according to sex and age group by weighting the underlying cause of death data from the Japanese national vital statistics in 2008 with multiple cause of death data from publically available US national vital statistics in 2006 (see Appendix).^13^ The equations, (1)–(3), are as follows: *P_JAPAN_*_,_*_j_*(*G*20|*D_i_*) is the proportion of death certificates mentioned with PD to those for *D_i_* as the underlying cause of death from disease *i* in the *j*-th sex-age-group in Japan; *D_i_* is ischemic heart disease (ICD-10: I20–I25), malignant neoplasm (C00–C75), cerebrovascular disease (I60–I69), pneumonia (J10–J18), PD (G20), or other (all other codes). The same is true for *P_US_*_,_*_j_*(*G*20|*D_i_*). *P_JAPAN_*_,_*_j_*(*G*20|*D_i_*) = 1 when *D_i_* is G20. Otherwise, equation (1) can be used: (1) *P_JAPAN_*_,_*_j_*(*G*20|*D_i_*) = $P_{US,j}(G20|D_{i})\times \frac{R_{JAPAN,j}}{R_{US,j}}$, where *R_JAPAN_*_,_*_j_* and *R_US_*_,_*_j_* are the death rates from PD as the underlying cause of death in the *j*-th sex-age-group in Japan and the United States, respectively. Equation (1) is used to adjust for the difference in disease structure between the 2 countries.

Then, the number of multiple-cause deaths from PD in the death certificate with *D_i_* as the underlying cause of death in the *j*-th sex-age-group, *M_i_*_,_*_j_*, is estimated as: (2) *M_i_*_,_*_j_* = *N_i_*_,_*_j_* × *P_JAPAN_*_,_*_j_*(*G*20|*D_i_*), where *N_i_*_,_*_j_* is the number of the underlying cause of death from *D_i_* in the *j*-th sex-age-group. The number of the multiple cause of death from PD in the *j*-th sex-age-group, *M_j_*, is obtained by summing *M_i_*_,_*_j_* for all underlying causes of death as: (3) $M_{j}=\sum_{i}M_{i,j}$. The sex- and age-specific rates of multiple-cause of deaths from PD were calculated by dividing *M_j_* by the corresponding sex- and age-specific Japanese population for the year 2008. The multiple cause of death rates, by sex and in total, were calculated from the corresponding numbers of summed multiple-cause deaths and populations.

## RESULTS

Mean age at death among PD decedents was 79.5 years for men, which exceeded the average life expectancy of men in the general population (79.3 years), and 82.0 years for women, which was lower than that of women in the general population (86.1 years; Table 1).

Table 2 shows all causes or conditions mentioned, other than PD, in the death certificates of 477 decedents. The 5 most frequent comorbid diseases listed as contributory causes of death were cerebrovascular diseases, dementia, diabetes mellitus, malignant neoplasm, and heart diseases. The most common immediate or intermediate cause of death was aspiration or suffocation that caused pneumonia, respiratory failure, or multiple organ failure leading to death.

Table 3 shows the death rates per 100 000 population according to underlying and multiple cause of death. Both rates increased with age, from 0.5 and 0.7, respectively, in those aged 55 to 64 years to 40.8 and 71.3 in those aged 85 years or older. The overall death rate estimated from extrapolation of US data on multiple-cause deaths was approximately 1.6 times that obtained from Japanese data on underlying cause of death: 5.8 versus 3.6, respectively.

**Table 2.** All causes or conditions mentioned, other than Parkinson’s disease, in death certificates of 477 decedents randomly selected from 4589 deaths due to Parkinson’s disease as the underlying cause of death (Japan, 2008)

Immediate causes^a^	Number	%	Intermediate causes^b^	Number	%	Contributory causes^c^	Number	%
Aspiration or suffocation	106	22.2	Aspiration or suffocation	64	13.4	Cerebrovascular diseases	19	4.0
Pneumonia	70	14.7	Senile deterioration	8	1.7	Dementia	18	3.8
Respiratory failure	61	12.8	Pneumonia	8	1.7	Diabetes mellitus	17	3.6
Senile deterioration	52	10.9	Dementia	5	1.0	Malignant neoplasms	12	2.5
Heart diseases	30	6.3	Cerebrovascular diseases	4	0.8	Heart diseases	11	2.3
Multiple organ failure	12	2.5	Respiratory failure	4	0.8	Lung diseases	10	2.1
CO_2_ narcosis or hypoxemia	9	1.9	Lung diseases	3	0.6	Infection, sepsis or DIC	8	1.7
Infection, sepsis or DIC	5	1.0	Heart diseases	3	0.6	Hypertension or hypotension	8	1.7
Renal diseases	3	0.6	Neuroleptic malignant syndrome	2	0.4	Diseases of the gastrointestinal tract	8	1.7
Lung diseases	2	0.4	Mental disorders	2	0.4	Fracture	6	1.3
Neuroleptic malignant syndrome	2	0.4	Infection, sepsis or DIC	2	0.4	Connective tissue diseases	6	1.3
Cerebrovascular diseases	2	0.4	Disuse syndrome	2	0.4	Diseases of arteries or arterioles	4	0.8
Diseases of the gastrointestinal tract	1	0.2	Diseases of the gastrointestinal tract	2	0.4	Senile deterioration	4	0.8
Disuse syndrome	1	0.2	CO_2_ narcosis or hypoxemia	2	0.4	Pneumonia	4	0.8
Unknown	1	0.2	Renal diseases	1	0.2	Mental disorders	4	0.8
			Hypertension or hypotension	1	0.2	Lung diseases	4	0.8
			Diabetes mellitus	1	0.2	Disuse syndrome	4	0.8
			Decubitus ulcer	1	0.2	Aspiration or suffocation	3	0.6
			Anemia or hypoalbuminemia	1	0.2	Liver diseases	3	0.6
						Decubitus ulcer	3	0.6
						Respiratory failure	1	0.2
						Anemia or hypoalbuminemia	1	0.2

Abbreviation: DIC, disseminated intravascular coagulation.

^a^An immediate cause is a final disease or condition resulting in death, described on the top line in Part (I) of the death certificate.

^b^Intermediate causes are diseases, injuries, or complications, other than immediate causes, in the chain of events that directly cause death, as described in Part (I) of the death certificate.

^c^Contributory causes are other significant conditions that contribute to the causes cited in Part (I), but do not directly result in those causes; they are described in Part (II) of the death certificate.

**Table 3.** Number and rate of Parkinson’s disease deaths, based on national vital statistics, by sex and age group (Japan, 2008): Underlying cause of death versus multiple cause of death

Age (years)	Underlying cause of death						Multiple cause of death^a^					
	
	Number of deaths			Rate per 100 000 ​population			Estimated number of deaths			Estimated rate per 100 000 ​population		
			
	Men	Women	Total	Men	Women	Total	Men	Women	Total	Men	Women	Total
0–14	0	0	0	0.0	0.0	0.0	0	0	0	0.0	0.0	0.0
15–24	0	0	0	0.0	0.0	0.0	0	0	0	0.0	0.0	0.0
25–34	0	0	0	0.0	0.0	0.0	0	0	0	0.0	0.0	0.0
35–44	2	1	3	0.0	0.0	0.0	2	2	4	0.0	0.0	0.0
45–54	6	7	13	0.1	0.1	0.1	9	10	19	0.1	0.1	0.1
55–64	53	32	85	0.6	0.3	0.5	83	45	128	0.9	0.5	0.7
65–74	381	297	678	5.4	3.7	4.5	623	407	1030	8.8	5.1	6.9
75–84	1187	1214	2401	29.4	21.2	24.6	2023	1769	3792	50.1	30.9	38.8
85+	495	914	1409	52.4	36.4	40.8	911	1549	2461	96.5	61.8	71.3
Total	2124	2465	4589	3.4	3.8	3.6	3652	3782	7434	5.9	5.8	5.8

^a^The estimated number and rate of multiple-cause PD deaths were calculated according to sex and age group by weighting data on underlying cause of death from 2008 Japanese national vital statistics with multiple cause of death data from publically available 2006 US national vital statistics (see reference 13).

**Appendix.** Number and rate of deaths with Parkinson’s disease mentioned on death certificates, by sex and age group (United States, 2006): Underlying cause of death versus multiple cause of death

Age (years)	Underlying cause of death						Multiple cause of death					
	
	Number of deaths			Rate per 100 000 ​population			Number of deaths			Rate per 100 000 ​population		
			
	Men	Women	Total	Men	Women	Total	Men	Women	Total	Men	Women	Total
0–14	0	1	1	0.0	0.0	0.0	0	2	2	0.0	0.0	0.0
15–24	0	1	1	0.0	0.0	0.0	0	0	0	0.0	0.0	0.0
25–34	0	0	0	0.0	0.0	0.0	3	0	0	0.0	0.0	0.0
35–44	4	1	5	0.0	0.0	0.0	4	4	0	0.0	0.0	0.0
45–54	36	16	52	0.2	0.1	0.1	67	30	97	0.3	0.1	0.2
55–64	251	130	381	1.7	0.8	1.2	450	239	689	3.0	1.5	2.2
65–74	1512	783	2295	17.4	7.6	12.1	2677	1359	4036	30.9	13.3	21.3
75–84	5517	3544	9061	104.1	45.7	69.5	9665	6147	15 812	182.4	79.3	121.2
85+	3894	3741	7635	230.7	103.7	144.1	6860	6809	13 669	406.3	188.7	258.1
Total	11 214	8217	19 431	7.6	5.4	6.5	19 726	14 590	34 316	13.4	9.6	11.5

Note: The data source is multiple cause of death data from publically available US national vital statistics (see reference 13).

## DISCUSSION

### Death certificate analysis

Our most noteworthy finding was that dementia and diabetes mellitus appeared together with the 3 leading causes of death—cerebrovascular disease, malignant neoplasm and heart disease—as comorbid diseases in a representative sample of the Japanese PD population. Because our analysis was able to detect chronic diseases and conditions that, while not fatal by themselves, could contribute to causing death, diabetes mellitus was highly ranked in our death certificate analysis, even though it is not among the 10 leading causes of death in the underlying-cause coding system of Japanese vital statistics. Much the same was true for dementia. Dementia is a major long-term cause of disability in people with PD and is reported in 30% to 80% of individuals with PD.^14^ The value observed in the present study, 4.8%, was quite low. This difference between studies may be due to differences in methods. The high prevalence of dementia in PD patients was mostly reported in clinical studies of PD patients who had undergone comprehensive neuropsychological assessment. Oral health, which is important in PD,^15^ was not mentioned anywhere in the death certificates of the present study.

Death certificate analysis has limitations. First, there is no multiple-cause coding system in Japan. Therefore, it was impossible to obtain death certificates in which PD was mentioned, but was not the underlying cause of death, from the 1.14 million death certificates filed in 2008. It may also be that parts of the analyzed death certificates were incomplete or inaccurate because of the possibility that (1) contributory causes are disregarded in data on underlying cause of death in national mortality statistics, (2) detailed medical records are sometimes unavailable at death, especially for decedents with long periods of morbidity, and (3) physicians sometimes have difficulty in reporting detailed medical information on the death certificates of such decedents.

### Analysis of vital statistics

The present study showed that analysis restricted to data on underlying cause of death underestimates PD mortality in Japan. Our estimated number of PD decedents was nearly 60% higher than the number of decedents due to PD as the underlying cause of death.

In the equation used in the present study, the estimates were adjusted for differences in disease structure between the United States and Japan. However, this adjustment could not fully account for racial differences between populations in vulnerabilities and comorbidities regarding PD. Age-standardized PD prevalence and incidence are lower in Japanese studies than in US studies.^16^ The underlying cause of death as a percentage of multiple cause of death reports was 49% in the US population in 2000–2001 and 56% in the UK population in 2001–2006.^4^^,^^10^ It remains to be seen whether these percentages are accurate for the Japanese population.

### Epidemiological implications

To estimate more accurately the national burden of PD comorbidity and mortality, a multidimensional approach is also required for the Japanese population. This approach is not a substitute for, but rather an extension of, existing data on underlying cause of death,^5^^,^^17^^,^^18^ and has already been adopted in the United States, the United Kingdom, Sweden, Spain, and France.^17^^–^^21^ The addition of this multiple-cause coding system to the current Japanese system would be an ideal long-term solution. It should also be recognized that, in a multiple-cause coding system, certain epidemiological catchment areas should be designated for the regular collection and release of necessary reference data on comorbidity and mortality statistics. Most importantly, it is essential to maintain the quality of death certificates by enhancing understanding of their importance in the fields of medicine, public health, and health policy.^5^^–^^7^

### Conclusion

The present study showed that analysis using only data from the underlying-cause coding system underestimated the national burden of PD comorbidity and mortality. Use of death certificates and multiple-cause mortality data are thus desirable complements to the existing system.
